# A Single-Center Retrospective Comparative Study of Conventional and Accelerated Fractionated Radiotherapy for Early-Stage Laryngeal Cancer

**DOI:** 10.7759/cureus.90693

**Published:** 2025-08-21

**Authors:** Juno Kaguchi, Kensei Nakata, Fumiyuki Suzuki, Masanori Someya, Hikaru Ikeda

**Affiliations:** 1 Department of Radiation Oncology, Sapporo City General Hospital, Sapporo, JPN; 2 Department of Otorhinolaryngology and Thyroid Surgery, Sapporo City General Hospital, Sapporo, JPN; 3 Department of Radiology, Division of Radiation Oncology, Sapporo Medical University, Sapporo, JPN; 4 Department of Radiation Oncology, Sapporo Higashi Tokushukai Hospital, Sapporo, JPN

**Keywords:** accelerated fractionation radiotherapy, altered fractionation radiotherapy, conventional radiotherapy, laryngeal cancer, moderate hypofractionation

## Abstract

Introduction: Radiotherapy is widely used in the treatment of early-stage laryngeal cancer (stage I and II). We analyzed the efficacy of conventional and accelerated fractionated radiotherapy (52.5 Gy in 16 fractions) for early-stage laryngeal cancer retrospectively.

Materials and methods: We compared the outcomes of 25 patients treated with conventional fractionation (CF group: 66-70 Gy in 33-35 fractions) and 20 patients treated with accelerated fractionation (AF group: 52.5 Gy in 16 fractions) radiotherapy from 2011 to 2024 at our institution.

Results: Local control rate was 87.8% at five years in the CF group and 95% at five years in the AF group. Acute adverse events were significantly higher in the CF group, with 72% Grade 2 or higher dermatitis compared to 20% in the AF group (p < 0.01). No serious late adverse events of Grade 2 or higher were observed in either group. The COVID-19 pandemic increased the use of AF from 34.3% before COVID-19 to 66.7% afterwards, suggesting that AF is a reasonable option as a curative strategy; AF has a shorter overall treatment time (median 22 days), better local control rates, and fewer adverse events.

Conclusion: Accelerated fractionated radiotherapy of 52.5 Gy in 16 fractions for early-stage laryngeal cancer is a safe and effective option for patients with poor performance status, older age, severe comorbidities, or difficulty with long-term hospital stays, offering a shorter overall treatment time without affecting the cure rate.

## Introduction

Laryngeal cancer is a common malignancy of the head and neck, and early-stage laryngeal cancer (stage I and II) is typically treated with radiotherapy because of its excellent local control and preservation of vocal function [1]. Conventional fractionated radiotherapy (CF), usually delivered over six to seven weeks, has long been the standard of care. However, there is increasing recognition that prolonged overall treatment time (OTT) may allow tumor cell repopulation, potentially compromising local control [2,3]. To address this concern, altered fractionation (AF) strategies have been developed. AF, which delivers higher doses per fraction over a shorter period, has become a widely adopted and clinically practical alternative, particularly for early-stage glottic cancer [2-13]. Studies have demonstrated that regimens such as 52.5 Gy in 16 fractions achieve comparable or superior local control to CF, with acceptable toxicity profiles [9-11]. Furthermore, the COVID-19 pandemic has underscored the value of shorter radiotherapy schedules in minimizing patient exposure and healthcare burden.

Although AF has already been in broad clinical use, direct comparisons between CF and AF are still relatively limited, particularly in terms of long-term outcomes and in real-world institutional settings. Differences in patient characteristics, tumor factors, and institutional protocols may influence outcomes, and further data from clinical practice are needed to inform treatment decisions. In this retrospective study, we aimed to compare the clinical outcomes of patients with early-stage laryngeal cancer treated with either CF or AF (52.5 Gy in 16 fractions) at our institution, in order to contribute to the evidence base supporting optimal fractionation strategies in this patient population.

## Materials and methods

Study design and setting

This was a retrospective, single-institutional study conducted at Sapporo City General Hospital to compare CF and AF radiotherapy regimens in patients diagnosed with early-stage laryngeal cancer.

Study period

Patients who underwent treatment between April 2011 and January 2024 were included in this study. The data collection and follow-up were completed by the cutoff date of October 2024.

Ethical approval

The study protocol was reviewed and approved by the institutional ethics committee (approval number R05-063-1082). As this was a retrospective study, an opt-out consent method was used in accordance with institutional and national ethical guidelines. Clinical data were anonymized prior to analysis.

Patient selection

Patients were included in the study if they met the following criteria: histologically confirmed squamous cell carcinoma of the larynx, clinical stage T1 to T2N0M0 according to the 8th edition of the American Joint Committee on Cancer (AJCC) staging system, and definitive treatment with radiotherapy at our institution. All patients underwent standard diagnostic workup including laryngeal fiberscopy, and head and neck imaging with computed tomography (CT) and/or magnetic resonance imaging (MRI).

**Figure 1:**
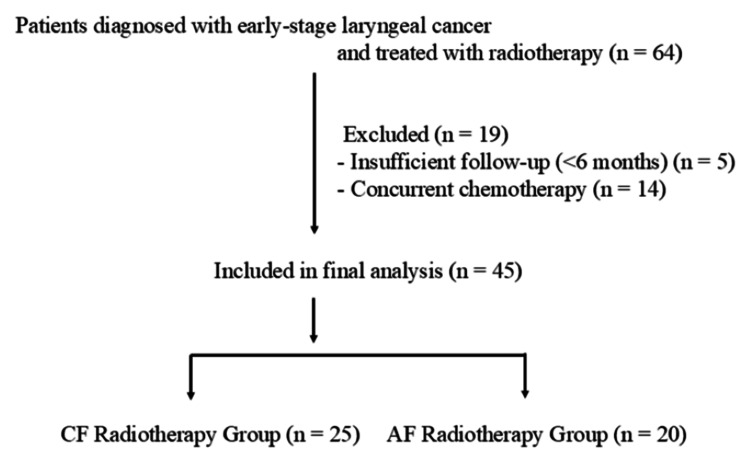
Flowchart of patient selection CF: conventional fractionation, AF: accelerated fractionation

Patients were excluded if they had previously received radiotherapy or surgery in the head and neck region, had evidence of nodal or distant metastasis, received concurrent chemotherapy, or had an incomplete course of treatment or insufficient follow-up duration (less than six months). A flowchart of patient selection is shown in Figure 1.

Treatment protocol

**Figure 2:**
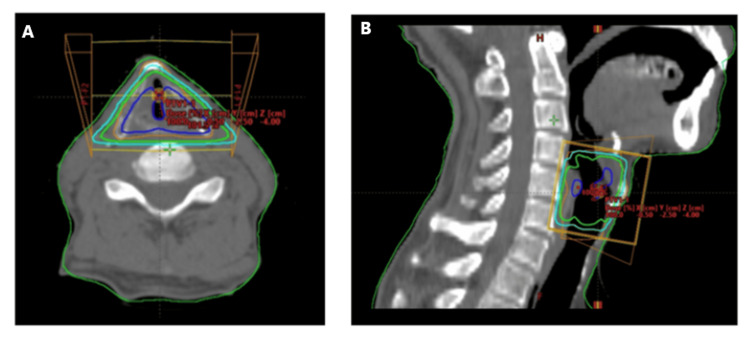
Dose distribution for a representative case. (A) Axial image, (B) Sagittal image. Blue: 100%, Orange: 95%, Green: 90%, and Light blue: 80% isodose line.

All patients received curative-intent radiotherapy using linear accelerators (CLINAC 600CD or CLINAC iX; Varian, Palo Alto, CA, USA) with either 4 MV or 6 MV X-rays. Clinical target volume adequately included the vocal cords and the thyroid cartilage. A lateral parallel-opposed field technique was employed, with field sizes generally ranging from 5 × 5 cm to 7 × 6 cm. A wedge filter was used to improve dose homogeneity across the treatment field. Treatment planning was performed using the Eclipse system (Varian) with the Analytical Anisotropic Algorithm (AAA) for dose calculation. A representative dose distribution is shown in Figure 2.

Patients in the CF group were treated with a total dose of 66 to 70 Gy delivered in 33 to 35 fractions over approximately 6.5 to seven weeks. Patients in the AF group received 50 to 52.5 Gy in 16 fractions over approximately three weeks. The decision to use AF radiotherapy was made through a shared decision-making process between the attending radiation oncologist and the patient. Factors influencing the choice of AF included advanced age, poor performance status (PS), comorbid conditions, and difficulty attending prolonged treatment courses. During the study period, several radiation oncologists determined the applicability of AF in each case.

Study parameters and outcome measures

Tumor response was assessed during follow-up visits using a combination of physical examination, fiberscopy, and imaging studies such as CT or MRI. Serum tumor markers (SCC, CYFRA, etc.) were also evaluated where appropriate. Patients were followed at intervals of one to two months during the first two years and at intervals of three to six months thereafter.

Overall survival (OS) was defined as the time from the start of radiotherapy to death from any cause or the date of last follow-up. Local control rate (LCR) was defined as the time from the end of radiotherapy to the date of local recurrence or last follow-up.

Adverse events were assessed according to the Common Terminology Criteria for Adverse Events (CTC-AE) version 5.0. Events occurring within three months after completion of radiotherapy were considered acute, while those occurring after three months were classified as late adverse events. Acute adverse events were assessed at least once a week during the radiotherapy and late adverse events were assessed once every three months. In addition, the amount and duration of opioid use during treatment were documented and used as an objective indicator of the severity of mucositis.

Dosimetric calculations

To compare the biological effects of different fractionation regimens, the biologically effective dose (BED) was calculated using the linear-quadratic model according to the formula BED (α/β) = n*d {1 + [d / (α/β)]}, where n is the number of fractions and d is the dose of a single fraction [14].

We used α/β = 10 to evaluate tumor response and acute adverse events, and α/β = 3 to evaluate late adverse events. To further convert to the normal fraction of 2 Gy, equivalent dose in 2Gy fractions (EQD2) was calculated based on BED as in the following equation: EQD2 = n*d (d + α/β) / (2 + α/β).

In radiotherapy for squamous cell carcinoma, a one-day prolongation of the treatment period is reported to decrease the therapeutic effect by the equivalent of 0.6 Gy because of accelerated repopulation that occurs during the treatment period [14]. Corrected EQD2 (cEQD2), which corrects for the treatment effect of prolonged OTT, is calculated by the following equation: cEQD2 = EQD2 - 0.6*(OTT - 28) [15].

For example, for 52.5 Gy in 16 fractions and a total treatment period of 22 days, BED10 = 69.7 Gy, BED3 Gy = 109.9 Gy, cEQD2 = 61.7 Gy.

Statistical analysis

Descriptive statistics were used to summarize patient demographics and treatment characteristics. OS and LCR were estimated using the Kaplan-Meier method, and comparisons between groups were made using the log-rank test. Fisher’s exact test was used to assess differences in variables between the CF and AF groups. A p-value less than 0.05 was considered statistically significant. Statistical analyses were performed using EZR software, version 1.33 (Saitama Medical Center, Saitama, Japan).

## Results

Patient characteristics are shown in Table 1. The CF group was significantly younger than the AF group (median age 69 years vs. 75 years, P = 0.03).

**Table 1:** Patients characteristics Median values are shown in parenthesis, CF: Conventional fractionation, AF: Accelerated fractionation, PS: Performance status, Fr: Fractions, OTT: Overall treatment time

	CF group (N=25)	AF group (N=20)	p value
Age (Years)	53 – 82 (69)	45 – 88 (75)	0.03
Gender			
Male	23 (92%)	19 (95%)	1
Female	2 (8%)	1 (5%)	
PS			
0 – 1	24 (96%)	15 (75%)	0.07
2 – 3	1 (4%)	5 (25%)	
Subsite			
Glottic	23 (92%)	17 (85%)	0.64
Supraglottic	2 (8%)	3 (15%)	
T stage			
T1a	12 (48%)	11 (55%)	0.76
T1b	8 (32%)	2 (10%)	
T2	5 (20%)	7 (35%)	
Radiation dose			
70 Gy/35 Fr	5 (20%)		
66 Gy/33 Fr	20 (80%)		
52.5 Gy/16 Fr		19 (95%)	
50 Gy/16 Fr		1 (5%)	
OTT (days)	44 – 52 (49)	21 – 28 (23)	< 0.01

**Figure 3:**
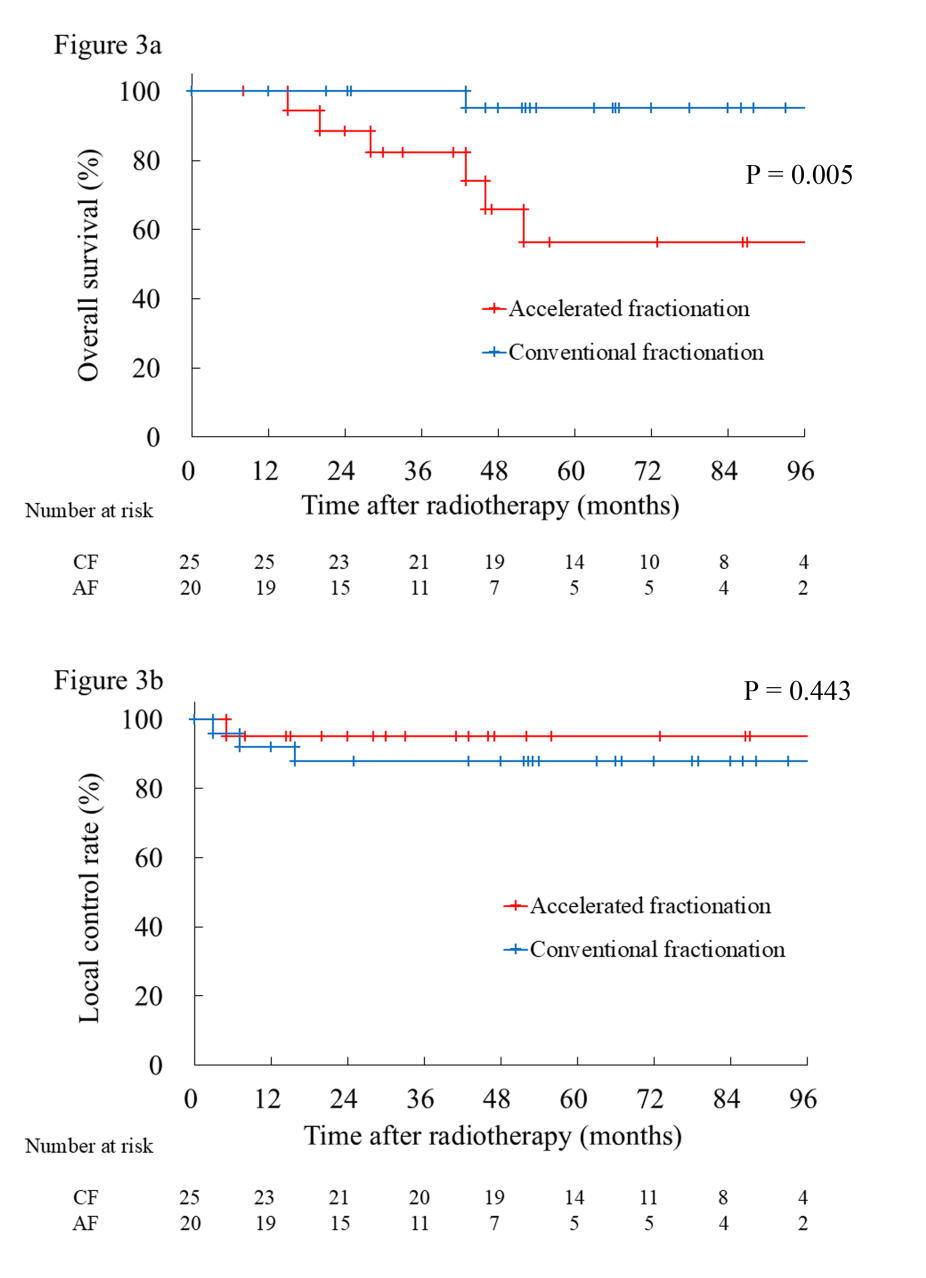
Outcomes according to fractionation schedule. (a) Overall survival (OS) was 100% at three years and 95.2% at five years in the CF group and 81.9% at three years and 56.2% at five years in the AF group (p = 0.005). (b) Local control rate (LCR) was 87.8% at three years and 87.8% at five years in the CF group, and 95% at three years and 95% at five years in the AF group (p = 0.443). CF: Conventional fractionation, AF: Accelerated fractionation

At the time of analysis, the median follow-up period was 42 months (range 8 - 120 months) for the AF group and 66 months (range 12 - 132 months) for the CF group, with three cases of local recurrence (CF group), one case of regional lymph node recurrence (AF group), and seven cases of death from other diseases (one in the CF group and six in the AF group; details shown in Table 2); salvage surgery was performed for the two cases of local recurrence. The median age of patients who died from other diseases was 78 years old at the time of beginning treatment, ranging from 69 to 88 years old. Compared to the CF group, the AF group had significantly poorer OS. OS was 100% at three years and 95.2% at five years in the CF group and 81.9% at three years and 56.2% at five years in the AF group (Figure 3A, p = 0.005). LCR was 87.8% at three years and 87.8% at five years in the CF group, and 95% at three years and 95% at five years in the AF group (Figure 3B, p = 0.443).

**Table 2:** Details of death from other disease. COPD: Chronic Obstructive Pulmonary Disease, GI: Gastrointestinal tract, CF: Conventional fractionation, AF: Accelerated fractionation

	CF group	AF group	Time after radiotherapy
	(N = 1)	(N = 6)	(months)
Renal failure	1	2	43, 20, 52
COPD	0	1	15
Lung cancer	0	2	28, 43
Upper GI bleeding	0	1	46

With regard to acute adverse events, Grade 2 or higher dermatitis was significantly more common in the CF group (72%) than in the AF group (20%) (p < 0.01). Grade 2 or higher mucositis was 68% in the CF group and 80% in the AF group, with no significant difference (p = 0.50). No Grade 2 or higher severe late adverse events were observed (Table 3).

**Table 3:** Acute and late adverse events. CF: Conventional fractionation, AF: Accelerated fractionation

	CF group (N=25)	AF group (N=20)	p value
Acute			
Mucositis			
Grade 1	8 (32%)	4 (20%)	Grade 1 vs 2-3
Grade 2	17 (68%)	15 (75%)	0.5
Grade 3	0	1 (5%)	
Dermatitis			
Grade 1	7 (28%)	16 (80%)	Grade 1 vs 2-3
Grade 2	16 (64%)	4 (20%)	< 0.001
Grade 3	2 (8%)	0	
Late			
Dysphagia			
Grade 1	1 (4%)	2 (10%)	0.57
Hoarseness			
Grade 1	1 (4%)	0	1

Details of opioid use are summarized in Table 4. Oral opioids were administered for the pain of acute mucositis in three patients (11.1%) in the CF group and in five patients (25%) in the AF group. There was no significant difference in the dose of morphine, but there was a trend toward longer administration of opioids in the AF group (median value: 15 days vs. 27 days, p = 0.32), but this was not statistically significant.

**Table 4:** Details of opioid use for mucositis. Dose of opioid was converted to equivalent morphine hydrochloride. Median values are shown in parenthesis, CF: Conventional fractionation, AF: Accelerated fractionation

	CF group (N=25)	AF group (N=20)	p value
Opioid use	3 (12%)	5 (25%)	0.43
Maximum dose (mg)	11.3 – 26.3 (15)	10 – 45 (10)	0.96
Time to start using (day)	27 – 36 (28)	19 – 22 (20)	0.44
Duration of opioid use (day)	7 – 43 (15)	23 – 81 (27)	0.32

Before COVID-19 (April 2011-December 2019), 22 patients were in the CF group and 12 in the AF group (34.3%), whereas after COVID-19 (January 2020-January 2024), three patients were in the CF group and eight in the AF group (66.7%). There was a trend toward more selection of AF due to the COVID-19 epidemic.

## Discussion

Cancer cells are known to exhibit accelerated repopulation in the latter half of the radiotherapy and it has been suggested that this could be overcome by shortening the treatment period with accelerated fractionation [14]. On the other hand, a higher radiation dose per fraction is associated with an increase in the acute adverse events. We started radical radiotherapy for early-stage laryngeal cancer in 1977, employing the so-called “Christie method” (52.5 Gy in 16 divided doses for four weeks) originating from the Christie Hospital in Manchester, England, for all patients [16]. Since 2007, CF or AF radiotherapy has been employed according to the patient's age, PS, and complications in each case.

In various cancers, hypofractionated radiotherapy has been drawing attention to shorten the OTT in order to deal with issues such as aging, poor PS, and the need for managing both work and cancer treatment. In addition, with the recent COVID-19 pandemic, shortening the OTT of outpatient treatment has been recommended [17-19], and also in Japan, the usefulness of hypofractionated radiotherapy has been recognized especially for breast and prostate cancers [20]. Similarly, the significance of hypofractionated radiotherapy for early-stage laryngeal cancer has been increasing. At our hospital, the number of patients treated with AF after the COVID-19 was increasing compared to that before the COVID-19 pandemic.

Although intensity modulated radiation therapy is now being attempted for the treatment of early-stage laryngeal cancer [10,21,22], the typical parallel opposed lateral fields radiotherapy is still the standard method. Treatment outcomes and adverse events with different dose fractionations can be compared with those previously reported without being affected by recent advances in treatment technology. Thus, a literature survey comparing several radiotherapy protocols with different dose fractionations using standard radiotherapy methods was performed and summarized in Table 5.

**Table 5:** Literature review on fractionations and clinical outcomes for early stage glottic cancer NA: not assessed, bid: twice a day, BED: Biological equivalent dose, OS: Overall survival, LCR: Local Control Rate AE: Adverse events

Authors	year	Dose	Dose per	n	BED10	BED3	cEQD2	5yOS	5yLCR	Acute	Late
			fraction (Gy)							AE ≥ G3	AE ≥ G2
Garden et al. [4]	2003	70Gy/35fr	2	89	84	116.7	58.6	0.73	0.72	NA	NA
T2 tumor											
Yamazaki et al. [5]	2006	66Gy/33fr	2	89	79.2	110	55.8	0.87	0.77	0.09	0.1
		63Gy/28fr	2.25	91	83.3	121	55.8	0.88	0.92	0.08	0.07
Sakata et al. [6]	2008	55Gy/32fr/bid	1.72 (bid)	97	64.5	86.5	57.3	0.92	0.87	0.036	0.071
Moon et al. [7]	2013	66Gy/33fr	2	82	79.2	110	55.8	0.825	-	≥ G2 5%	0.02
		63Gy/28fr	2.25	74	83.3	121	55.8	0.866	-	≥ G2 1%	0
Trotti et al. [8]	2014	70Gy/35fr	2	119	84	116.7	58.6	0.63	0.704	0.125	0.314
T2 Tumor		79.2/33fr/bid	1.2 (bid)	120	88.7	110.9		0.72	0.781	0.175	0.328
Motegi et al. [9]	2015	64.8Gy/27fr	2.4	44	80.4	116.6	61.6	0.91	0.77	0	NA
T2 Tumor											
Berwouts et al. [10]	2015	70Gy/35fr	2	22	84	116.7	58.6	0.75	0.74	0.407	0.057
T2 Tumor											
Szutkowski et al. [11]	2016	66Gy/33fr	2	199	79.2	110	55.8	0.862	-	≥ G2 68%	0.09
		63Gy/28fr	2.25	223	83.3	121	55.8	0.875	-	≥ G2 52%	0.03
Kodaira et al. [12,13]	2022	66-70Gy/33-35fr	2	184	84	116.7	58.6	0.927	-	0.06	0.005
		60-64.8/25-27fr	2.4	186	80.4	116.6	61.6	0.896	-	0.051	0.005
Current study		52.5Gy/16fr	3.28	20	69.7	109.9	61.7	0.562	0.95	0.05	0
		66-70Gy/33-35fr	2	25	84	116.7	58.6	0.955	0.887	0.08	0

According to these studies, the OS of conventional fractionated radiotherapy for early-stage laryngeal cancer ranged from 63.0 to 92.7% at five years, and LCR ranged from 70.4 to 77.0% at five years, respectively. Moderate hypofractionated radiotherapy with a dose of 2.2 to 2.5 Gy per fraction, the OS ranged from 86.6 to 92.0% at five years, and the LCR ranged from 77.0 to 92.0% at five years, respectively [4,5,7,8,11,12]. In the present study, OS in the AF group was 56.2% at five years, and LCR was 95% at five years. The lower OS may be due to the fact that AF was selected for elderly patients and poor PS (as shown in Table 1), and there were six deaths from other diseases (Table 2), however, the LCR was comparable to the results with various dose fractionation.

The result of JCOG0701 has shown that acute grade 3 dermatitis in the CF group was 3.8% and mucositis was 6%, while in the moderate hypofractionated group, acute grade 3 dermatitis was reported in 3.8% and mucositis in 5.1% (2.4 Gy per fraction) [12]. In the JCOG0701A3 study, laryngeal edema, pharyngeal headache, soft tissue necrosis, sclerosis, and voice change were reported as late adverse events, but the incidence was reported to have shown no clear difference between each group [13]. In the AF group in this study, acute grade 3 dermatitis was observed in 0%, grade 3 mucositis in 5%, late grade 1 dysphagia in 10%, and grade 2 or higher of late adverse events were not observed. Compared with these results of CF and AF schedules, this treatment could be completed without enhancement of adverse events (Table 5). In addition, the rate, amount, and duration of opioid use used in this study as a surrogate for subjective assessment of acute mucositis showed no statistically significant differences between the CF and AF groups (Table 4).

With regard to acute adverse events, Grade 2 or higher dermatitis was significantly more common in the CF group (72%) than in the AF group (20%) (p < 0.01) in the current study.

A systematic review by Lee et al. reported that moderate-hypofractionated and ultra-hypofractionated radiotherapy significantly reduced the incidence of grade 2 or higher radiation dermatitis (risk ratio: 0.59) compared with CF in postoperative radiotherapy for breast cancer [23]. The biological effects of radiotherapy depend on dose per fraction and fraction size. While CF has a lower dose of 2 Gy per fraction, hypofractionated radiotherapy has relatively higher dose per fraction and smaller fraction size, which may increase the time available for tissue repair. Cumulative damage from repeated radiotherapy by CF may lead to the worsening of dermatitis, especially in acute responding tissues such as skin.

Table 5 shows the cEQD2, BED10, and BED3 for the several fractionation schedules used in radiotherapy for early-stage laryngeal cancer. A regimen of 52.5 Gy in 16 fractions has a higher tumor effect (cEQD2) and lower acute adverse events (BED10) and late adverse events (BED3) than conventional fractionation. The model equation also indicates that, similar to the clinical results, the treatment is expected to be more effective than other fractionation schedules without increasing the number of adverse events.

Important limitations of this study include, first, the relatively small sample size, which prevented performing multivariate analysis to adjust for multiple confounding factors. OS may be susceptible to being affected by confounding factors. In fact, in this study, a significant difference in OS was observed between the CF group and the AF group. Second, since the CF group and AF group were not randomly assigned, there may be potential selection bias, and heterogeneity may exist in patient profiles. These are limitations of a retrospective observational study conducted at a single institution. In addition, there are individual differences in susceptibility to opioids, and it may not be appropriate to evaluate acute mucositis based on a simple comparison of doses of opioids. Regarding late adverse events, the lack of accurate evaluation of voice function remains a problem, and further research should be performed on the reduction of late adverse events through the use of intensity modulated radiotherapy (IMRT). However, the irradiation technique and treatment plan in this study are simple and can be performed at any institution.

## Conclusions

This study demonstrated that accelerated fractionated radiotherapy (52.5 Gy in 16 fractions) is a safe and effective treatment option for early-stage laryngeal cancer. The regimen allows for a shorter overall treatment time without compromising local control.

AF may be particularly useful for patients who are elderly, have poor performance status, or face challenges with prolonged hospital visits. Given its clinical efficacy and convenience, this approach can serve as a practical alternative to conventional fractionation in appropriate patients.
